# Supplementary material for: The interactome of intact mitochondria by cross-linking mass spectrometry provides evidence for coexisting respiratory supercomplexes
Source: Mol Cell Proteomics. 2017 Dec 8;17(2):216–32. doi: 10.1074/mcp.RA117.000470 (PMC5795388; doi:10.1074/mcp.RA117.000470)
Supplement: Supplemental Data [file supp_RA117.000470_133922_0_supp_23978_fzffwf.zip › spectra_annotation/mito_spectra_annotation/452-1-5-1-11-1.pdf]

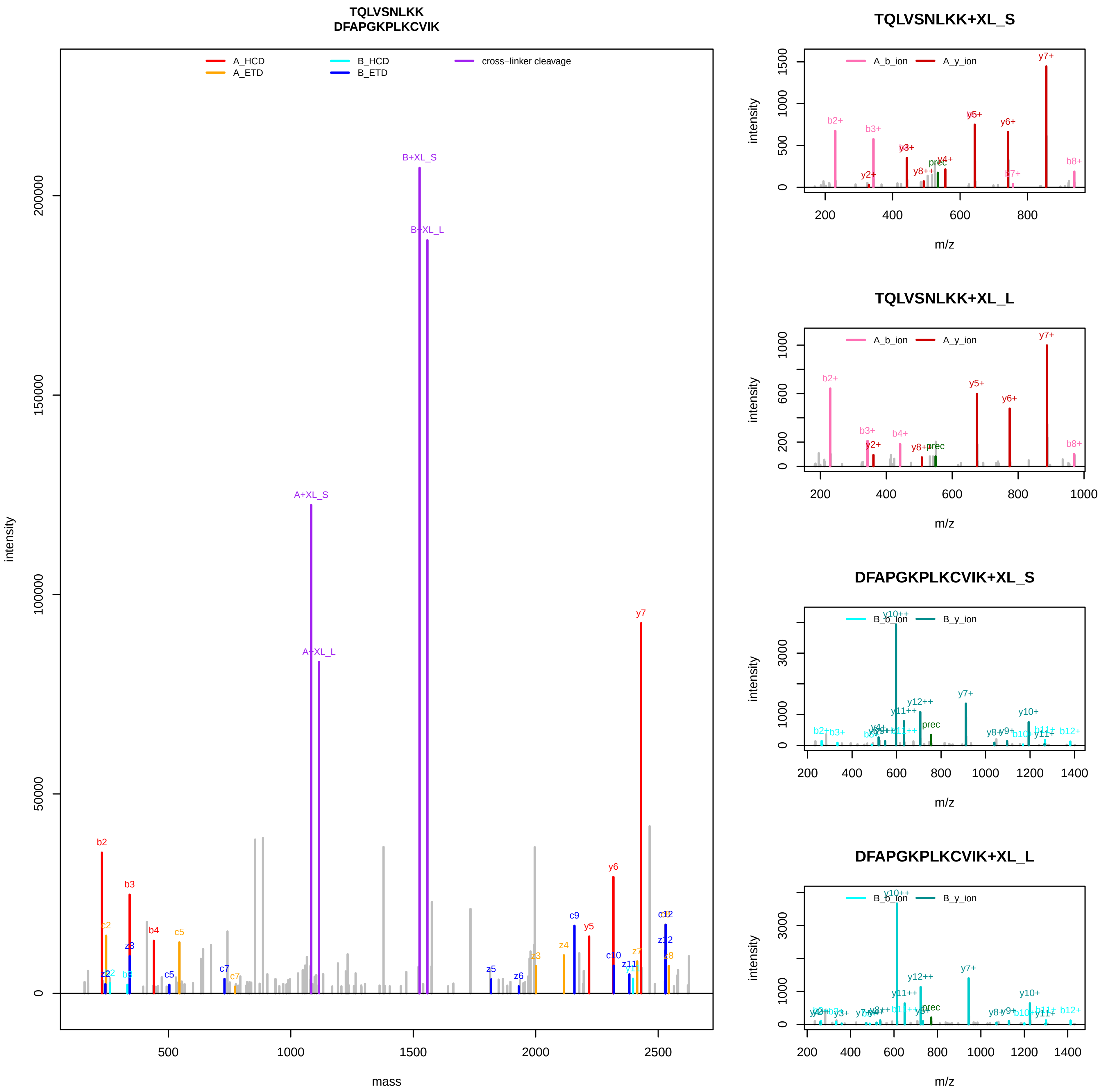

TQLVSNLKK
DFAPGKPLKCVIK
A_HCD
A_ETD
B_HCD
B_ETD
cross−linker cleavage
B+XL_S
B+XL_L
A+XL_S
A+XL_L
b2
b3
b4
c2
c5
c7
c9
c10
c12
z2
z3
z4
z5
z6
z7
z8
z11
z12
y5
y6
y7
intensity
mass
TQLVSNLKK+XL_S
TQLVSNLKK+XL_L
DFAPGKPLKCVIK+XL_S
DFAPGKPLKCVIK+XL_L
A_b_ion
A_y_ion
B_b_ion
B_y_ion
prec
b2+
b3+
b4+
b7+
b8+
b12+
y2+
y3+
y4+
y5+
y6+
y7+
y8++
y10+
y10++
y11+
y11++
y12++
m/z
